# Pyosalpinx complicating chronic hydrosalpinx in a 50-year old virgo woman: a case report

**DOI:** 10.1186/s12905-018-0583-3

**Published:** 2018-06-11

**Authors:** Yannick Hurni, Marta Bonollo, Ludovica Ferrero, Gianmarco Taraschi, Claudia Canonica, Sophie Venturelli Reyes Lozano

**Affiliations:** Department of Obstetric and Gynecology, Ospedale Regionale Bellinzona e Valli, 6500 Bellinzona, Switzerland

**Keywords:** Virgo, Pyosalpinx, Pelvic inflammatory disease

## Abstract

**Background:**

Pelvic inflammatory disease is an infection of the upper genital tract, including the uterus, ovaries, uterine tubes, and pelvic peritoneum. Tubo-ovarian abscess and pyosalpinx are common complications associated with pelvic inflammatory disease. They are usually encountered in sexually active women, but rare cases in Virgos have also been described.

**Case presentation:**

Here, we report the case of a 50-year-old Virgo woman presenting with pyosalpinx secondary to previous laparotomic sigmoidectomy for acute diverticulitis. Inflammation caused by the woman’s diverticulitis and laparotomic surgery could have been the origin of her left uterine tube occlusion and consequent hydrosalpinx development. The contact between the rectum and left uterine tube observed in our patient suggests that superinfection of the hydrosalpinx could have occurred secondary to bacterial translocation. The patient’s condition was managed with laparoscopic left salpingectomy and antibiotic therapy, which resulted in complete resolution.

**Conclusions:**

Regardless of sexual history, pelvic inflammatory disease should be considered in all women with abdominal pain. Diagnosing pelvic inflammatory disease in Virgos could be very challenging, but its recognition and appropriate treatment are indispensable because of the potential long-term complications.

## Background

Pelvic inflammatory disease (PID) is an infection of a woman’s reproductive organs, including the uterus, ovaries, uterine tubes, and pelvic peritoneum. Tubo-ovarian abscess and pyosalpinx are common complications associated with PID. They are usually encountered in sexually active women, but rare cases in Virgos have also been reported [1]. Here, we report a case of a Virgo woman with pyosalpinx developing from a pre-existing hydrosalpinx secondary to abdominal surgery.

## Case presentation

A 50-year-old Virgo woman in perimenopause presented to the emergency department with a 24-h history of intermittent abdominal pain and subfebrile body temperature up to 38 °C, associated with vaginal spotting for several days. She denied experiencing recent weight loss, diarrhea, vomiting, abnormal vaginal discharge, dysuria, or increased urinary frequency. She reported irregular menses with metrorrhagia in the last 12 months. The patient reported being a Catholic sister and denied having had past oral, vaginal, or anal intercourse.

**Fig. 1:**
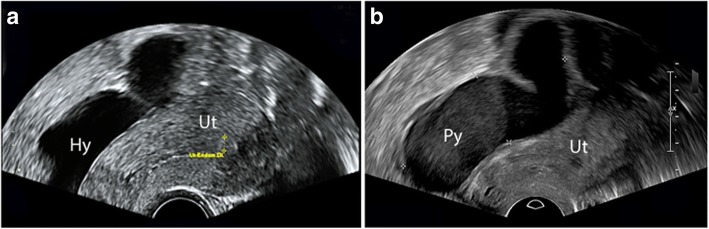
**a** Transvaginal ultrasound performed 3 months prior to patient admission during a routine gynecological visit, showing a tubular structure of 8.1 × 2.0 cm in size filled with anechoic fluid on the left side of the uterus and interpreted to be a hydrosalpinx. **b** Transvaginal ultrasound at admission, showing a tender tubular structure of 7.3 × 2.1 cm in size filled with low-level echogenic fluid in the left pelvis and interpreted to be a pyosalpinx. *Ut = uterus; Hy = hydrosalpinx; Py = pyosalpinx*

Her past medical history was significant for perforated acute diverticulitis of the sigmoid colon, which had been managed with laparotomic sigmoidectomy and antibiotic therapy 2 years earlier. Following this event, her gyneeologist observed the apparition of a left hydrosalpinx that was 8.1 × 2.0 cm in size and filled with anechoic fluid on ultrasound **(**Fig. 1a**)**. The patient appeared asymptomatic and no other relevant elements were found.

Upon presentation to the emergency room, the patient’s vital parameters were stable, and she was subfebrile (37.7 °C). A physical examination demonstrated bilateral lower abdominal quadrant tenderness without involuntary guarding or rebound pain. Blood analysis showed a white blood count of 10.9 × 10^9^/L (normal 4.0–10.0 × 10^9^/L) and C-reactive protein count of 128 mg/L (normal < 5 mg/L). A urine pregnancy test and urinalysis were both negative. An abdominopelvic rectal-contrast computed tomography scan excluded diverticulitis and other intestinal abnormalities, and revealed a left complex adnexal mass measured to be 7.5 × 4.4 cm in size. The patient was then referred to our gynecology department. A gynecological exam revealed the presence of bloody vaginal discharge with an elevated number of leukocytes upon microscopic exam. A vaginal ultrasound showed a dilated tubular structure 7.3 × 2.1 cm in size, filled with low-level echogenic fluid in the left pelvis **(**Fig. 1b**)**. The patient was presumed to have left pyosalpinx as a complication of hydrosalpinx superinfection, and she consented to an urgent exploratory laparoscopy. Preoperatively ceftriaxone IV (2 g) and metronidazole (500 mg) were given.

Upon entry, important adhesions in the entire abdominal cavity were noted. The small and large bowels were explored and appeared normal. A left pyosalpinx was noted and appeared to be firmly adherent to the uterus and rectum. During its liberation, unintentional pyosalpinx perforation occurred, and purulent drainage was noted. The fluid was sent for microbiologic and cytologic analysis. A left salpingectomy was performed, with consequent retrieval of the specimen in an endobag. Both ovaries appeared normal. The right uterine tube appeared slightly hyperemic, but pyosalpinx and tubo-ovarian abscess were excluded. A percutaneous drain was placed in the left pelvis. The abdomen was then irrigated with 2 L of 0.9% sodium chloride solution and closed.

Postoperatively, the patient received parenteral antibiotic therapy with ceftriaxone (2 g every 24 h) and metronidazole (500 mg every 8 h) until discharge on postoperative day three. Nucleic acid amplification to identify cervical gonorrhea or chlamydia had negative results. The pyosalpinx pus, vaginal swab, blood, and urine cultures were all negative, and empiric oral antibiotic therapy with metronidazole (250 mg every 8 h) and levofloxacin (500 mg every 12 h) was pursued. The cytologic and histologic analysis results were consistent with a diagnosis of pyosalpinx without other pathologic findings. The postoperative course was uneventful, and the patient was discharged home with gynecological follow-up 10 days later.

At the follow-up visit, the patient reported the near resolution of her pain, and no clinical, ultrasonographic, or laboratory abnormalities were found.

## Discussion and conclusions

Tubo-ovarian abscess and pyosalpinx are almost always complications of PID. Since sexual intercourse with an infected partner is the most critical predisposing factor, these conditions are rarely observed in Virgo patients. Other risk factors include an intrauterine device, previous history of PID, and multiple sexual partners. The typical organisms isolated in cases of PID include *Neisseria gonorrhea* and *Chlamydia trachomatis*, while *Bacteroides*, *Peptostreptococcus*, *streptococcal* species, *Escherichia coli*, and other gram-negative enteric organisms are commonly found in cases of tubo-ovarian abscess or pyosalpinx.

Until now, only 27 cases of PID in Virgos have been reported in the literature and were accurately summarized by Cho et al. [1]. Previous studies have suggested potential physiopathological mechanisms causing PID in Virgo women [1]. Among them, ascending infection from the lower genital tract is the most common hypothesis. This could be the result of vaginal infection resulting from local pathogens or contamination through prolonged contact with infected urine or stool. Therefore, urinary tract infection, poor hygiene, fecal incontinence, anogenital malformations, and pooling of urine in the posterior vagina secondary to any reason seem to be the principally described risk factors [1]. Another hypothesis proposed bacterial spread by hematogenous seeding, or direct translocation from the gastrointestinal tract, as the origin of infections. Tubo-ovarian abscess in Virgo patients who were concomitantly presenting Crohn disease, sigmoid diverticulits, bowel obstruction, or previous pelvic surgery for major bowel anomalies have been reported [2–6].

Similarly, we have described here the case of a Virgo woman presenting pyosalpinx secondary to previous laparotomic sigmoidectomy for acute diverticulitis. Inflammation caused by diverticulitis and laparotomic surgery could have been the origin of her left uterine tube occlusion and consequent hydrosalpinx development [7]. The firm contact between the rectum and left uterine tube observed during laparoscopy suggests that superinfection of the hydrosalpinx could have occurred secondary to bacterial translocation from the bowel. This is also supported by the fact that the serous liquid contained in the hydrosalpinx could be a gut medium for bacterial growth [8, 9]. Unfortunately, as in other reported cases, the cultures were negative probably due to previous antibiotic therapy. In other studies, the most common organism isolated in Virgo women was *Escherichia coli*, followed by *Streptococcus* species [1]. In our case, after discussion with infectious-disease specialists, typical antibiotic therapy directed against intestinal microorganisms with intravenous ceftriaxone and metronidazole was administered postoperatively.

This case highlights the importance of including PID during the differential diagnosis of acute abdomen in sexually inactive females. Regardless of sexual history, PID should be considered in all women with abdominal pain. Diagnosing PID in Virgos could be very challenging, but its recognition and appropriate treatment are indispensable because of the potential long-term complications, including risk of chronic pelvic pain, ectopic pregnancy and infertility. Particular attention should be paid to patients with recurrent urogenital infections, previous abdominopelvic surgery, uni- or bilateral hydrosalpinx, and inflammatory or infectious intestinal diseases.

## Abbreviations

PID: Pelvic inflammatory disease
